# Evolving roles of glycosylation in the tug-of-war between virus and host

**DOI:** 10.1093/nsr/nwae086

**Published:** 2024-03-07

**Authors:** Xiaofeng Zhai, Yanqiu Yuan, Wan-Ting He, Ying Wu, Yi Shi, Shuo Su, Quansheng Du, Yang Mao

**Affiliations:** Academy for Advanced Interdisciplinary Studies, Engineering Laboratory of Animal Immunity of Jiangsu Province, College of Veterinary Medicine, Nanjing Agricultural University, China; State Key Laboratory of Anti-Infective Drug Discovery and Development, School of Pharmaceutical Sciences, Sun Yat-sen University, China; School of Pharmacy, China Pharmaceutical University, China; Guangdong Provincial Key Laboratory of Drug Non-Clinical Evaluation and Research, School of Pharmaceutical Sciences, Sun Yat-sen University, China; CAS Key Laboratory of Pathogen Microbiology and Immunology, Institute of Microbiology, Chinese Academy of Sciences, China; Academy for Advanced Interdisciplinary Studies, Engineering Laboratory of Animal Immunity of Jiangsu Province, College of Veterinary Medicine, Nanjing Agricultural University, China; Department of Interdisciplinary Sciences, National Natural Science Foundation of China, China; Guangdong Provincial Key Laboratory of Drug Non-Clinical Evaluation and Research, School of Pharmaceutical Sciences, Sun Yat-sen University, China

Covering the surface of both host cells and enveloped viruses, glycans are inevitable biomolecules for investigating virus–host interactions. However, due to the intricate role of glycans in the viral–host relationship, few glycan-related molecular mechanisms have been harnessed for antiviral therapies. The intricacy arises from multiple factors. From the perspective of viral infection, host cell surface glycans are important anchoring molecules for virion binding and internalization. From the perspective of host defense, innate immune cells and their associated molecules recognize viral surface glycans and mark them for destruction [1,2]. Furthermore, enveloped viruses could hijack the glycosylation machinery of host cells to establish their own ‘glycan shield’, enabling them to evade immune detection. Concomitantly, host cells employ glycosylation to impede the activation of envelope proteins and the assembly of virions [1,2]. Glycan is such an essential weapon exploited by both viruses and hosts that a comprehensive understanding of glycan-associated mechanisms in viral infection and the immune defense of the host could facilitate the development of antiviral medicines and vaccines. In this short essay, we summarize the multifaceted function of glycans in virus–host interactions, along with challenges and future perspectives.

The host cell surface is covered by glycocalyx—a mesh-like structure composed of various glycoproteins, glycolipids and proteoglycans. It can reach hundreds of nanometers away from the plasma membrane and provide a physical barrier against invading viruses by shielding potential viral receptors (20–30 nm) [3] (Fig. 1). However, host-infecting viruses have evolved to exploit the cell surface glycan structures either as initial attachment factors or as entry receptors (Fig. 2A and Table S1). Therefore, the features of host surface glycans are crucial for determining viral pathogenesis and host tropism [2]. Mechanistically, virus–glycan interaction restricts virus mobility, immobilizes virion particles on the cell surface and eventually facilitates receptor-mediated internalization.

**Figure 1.**
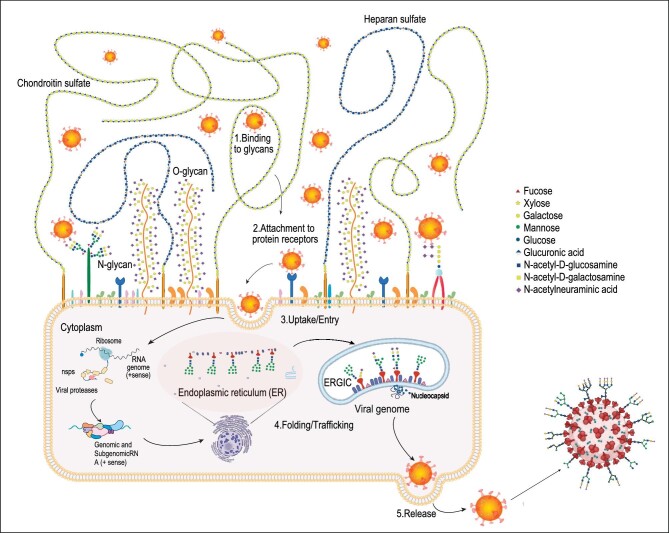
Glycans on both the host cell and the viral surface are involved in all steps of viral infection. During the initial binding, viruses preferentially attach to glycans on the host cell surface (1) and then interact with receptors on the cell surface (2). Certain glycans directly mediate viral entry as cell surface receptors (3). During viral protein trafficking in the endoplasmic reticulum (ER) and Golgi, glycosylation affects protein synthesis, folding and assembly (4). Glycans also play a role in virus release as in the case of neuraminidase-facilitated IAV release (5). The layout of surface glycalyx is adopted from the literature [3].

**Figure 2.**
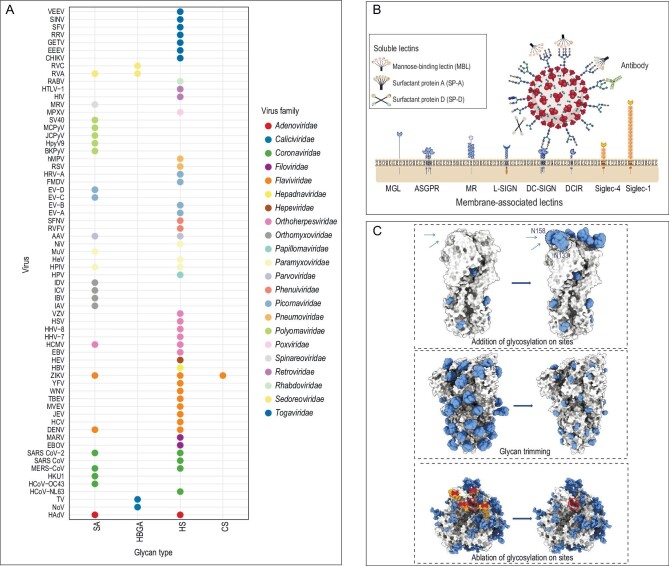
(A) Summary of major types of glycans on the host cell surface that can be recognized by various viruses in their initial attachment. HS, heparan sulfate; CS, chondroitin sulfate; HBGA, histo-blood group antigens; SA, sialic acid. (B) Interactions between glycosylated viral proteins and host cell factors, which include host lectin receptors, free lectins or antibodies [17]. MGL, macrophage Gal/GalNAc-specific C-type lectin; ASGPR, asialoglycoprotein receptor; MR, mannose receptor; L-SIGN, liver/lymph node-specific ICAM-3 grabbing non-integrin; DC-SIGN, dendritic cell-specific intracellular adhesion molecules (ICAM)-3 grabbing non-integrin; DCIR, dendritic cell immunoreceptor. (C) Glycoengineering-based vaccine design strategy. Structural models were generated by PyMOL(TM) 2.5.5 (influenza viruses: PDB 4LCX; Severe Acute Respiratory Syndrome Coronavirus 2: PDB 6VSB; Human Immunodeficiency Virus: PDB 5FYL) and shown in surface representation.

A typical example of the glycan-based attachment factor is glycosaminoglycans (GAGs), which are abundant linear polysaccharides made of 10–100 repeating disaccharide units with varying degrees of sulfation. The highly negatively charged nature of GAGs makes it a distinct chemical feature for viral attachment. As a result, many viruses, including those that have well-characterized internalization receptors, are reported to contain positively charged patches on their surface proteins, which bind to GAGs through electrostatic interactions. For example, previous mechanistic studies on HIV infection have demonstrated the direct binding between viral surface glycoprotein GP120 and cell surface heparan sulfate (HS) on permissive and non-permissive cells, which facilitates *in cis* and *in trans* capturing of the HIV virion and eventually leads to its recognition by protein receptor CD4 [4]. Similarly, recent studies on SARS-CoV-2 have revealed a dependence of viral infection on both HS as the attachment factor and ACE2 as the protein receptor. The binding of HS to the electropositive surface in the spike protein of SARS-CoV-2 promotes its interaction with ACE2, facilitating viral entry [5]. It should be noted that binding to GAGs does not always enhance infectivity. In the case of herpes simplex virus type 1 (HSV-1), while binding to HS promotes virus confinement and increases cellular entry, binding to chondroitin sulfate (CS), another type of GAG, promotes the free diffusion of the virus and hinders its attachment to cell surfaces [6]. The distinct roles of different GAGs in viral attachment are likely attributed to the specificity of molecular recognition and possibly the density of the binder partner between the virus and host glycocalyx.

Sialic acid (SA) is another well-known glycan structure that viruses exploit for initial attachment [7]. SAs belong to a family of negatively charged 9-carbon monosaccharides, which can be found at the termini of a variety of glycans, connected via α2,3, α2,6 or α2,8 linkages [7]. Influenza A virus (IAV) is a well-studied example of viruses using sialylated proteins and gangliosides as entry receptors. The IAV envelope protein hemagglutinin (HA) contains a lectin-like domain that is mainly responsible for SA recognition. It is generally accepted that HA proteins from human strains preferentially recognize α2,6-linked SAs while those from avian strains preferentially recognize α2,3-linked SAs [7]. This preferential interaction, mediated by the lectin-like domain of HA, thus differs from the electrostatic attraction-driven binding of HS mentioned above and is believed to serve as a species barrier between avian and human hosts of IAV. Although efforts have been made to develop anti-influenza therapeutics that block the interaction between HA and SAs, these research findings have not yet been translated into clinical applications. Aside from this classical example, SAs are recognized as attachment factors by a few other enveloped and non-enveloped viruses, such as reovirus, adenovirus, rotavirus, etc. (Fig. 2A). Interestingly, SARS-CoV-2 was recently reported to recognize SAs as well, in addition to its protein receptor ACE2 and glycan attachment factor HS [8]. SAs may serve a function similar to HS for SARS-CoV-2, which enriches virion particles on the cell surface and promotes viral interactions with ACE2.

On the virus side, due to the limited size of the viral genome, viruses lack their own glycosylation machinery. However, they acquire glycosylation by including potential glycosylation sites in their protein sequences and hijacking the secretory pathway of the host cells, which consists of a consortium of glycosyltransferases, glycosidases and other enzymes. From an evolutionary perspective, the question arises as to why enveloped viruses develop glycosylation sites in the first place. As *N*-linked glycosylation is typically used by host cells to facilitate protein folding, one obvious benefit for viruses to include *N*-glycosylation in their membrane protein is to increase its stability and structural integrity. Besides, glycans can be directly involved in modulating functions of modified viral proteins. As a notable example, two *N*-glycosylation sites in the N-terminal domain of the SARS-CoV-2 spike protein play essential functional roles in adjusting the conformation of the receptor binding domain of the spike [9]. Moreover, the incorporation of extensive glycans on the surface of viruses provides a protective ‘glycan shield’ for critical immunogenic epitopes, since viral glycans derived from host cells are generally recognized as ‘self’ by the host [1]. However, as the assembly of virion competes with the glycosylation process in host cells, virus surface proteins can display glycoforms atypical of normal host membrane proteins. These atypical glycoforms are recognized by innate immune molecules such as collectins and elicit immune responses [10] (Fig. 2B). For example, the gp120 envelope glycoprotein of HIV-1 is densely coated with an immature high-mannose type of N-glycans, which form an intrinsic mannose patch on the surface of HIV and serve as epitopes for a number of broadly neutralizing antibodies [11]. On the other hand, viruses also evolved to take advantage of those lectin receptors to gain entry into host (immune) cells (summarized in Table S2). In the case of HIV-1, recognition of the high-mannose glycans on gp120 by dendritic cell-specific C-type lectins (DC-SIGN) is exploited by the virus to adhere to dendritic cells and to enhance its infection of target cells *in trans* [12].

Importantly, the ‘glycan shield’ that helps viruses to evade immune recognition can also influence the interaction between the virus and protein receptors on host cells, thereby affecting infectivity and virulence [1]. Consequently, the number and location of glycosylation sites on the virus surface could reflect a balance between immune suppression and preservation of viral membrane protein functions. Repositioning of glycosylation sites on virus membrane protein thus indicates that the virus breaks out of immune surveillance by masking existing neutralizing epitopes with altered glycosylation [13]. In addition, recent findings indicate that mucin-type *O*-glycosylation, which modifies serine and threonine residues near the convertase-processing site of the spike protein of SARS-CoV-2, negatively regulates virion activation [14]. The specific sites of these *O*-linked glycosylation are determined by the substrate specificities of host cell *O*-GalNAc transferases, reflecting the ability of the host cells to utilize their own glycosylation machinery to inhibit viral activity. Interestingly, recent studies have found that many structural and non-structural viral proteins can be modified with *O*-GlcNAcylation—a wide-spread form of intracellular monoglycosylation (summarized in Table S3). Host cells might utilize this glycosylation form to restrain the replication of the viral genome, which is an interesting topic worth further investigation.

As the glycosylation of viral surface proteins plays important roles in modulating immune recognition and epitope exposure, it is a critical factor to be considered in the design of vaccines (Table S4). Firstly, the presence of glycosylation influences the choices of host cells for vaccine production. Different host cells harbor different sets of glycosylation enzymes, thereby affecting the glycoforms present on produced vaccines and consequently the biological activities. This is an important consideration, especially when developing a recombinant protein-based vaccine that aims to mimic the native glycosylation state of authentic viruses. For example, current influenza vaccines are predominantly produced in embryonated chicken eggs, resulting in complex-type glycans on surface glycoproteins. However, FluBlok, the currently available recombinant HA protein vaccine, is expressed in insect cells and contains paucimannose- and high-mannose-type glycans [15]. A recent comparative study found that FluBlok elicited higher influenza-specific antibody responses compared with vaccines produced in chicken eggs or mammalian cells, which may be attributed to variations in glycosylation [15]. Secondly, as the hyper-glycosylation of viral surface glycoproteins reduces host immune responses, tailor-designed glycoengineering has been explored as a novel strategy in recent vaccine designs (Fig. 2C). In the most straightforward way, by reducing the size of glycans on virus envelope proteins and exposing protected immunogenic epitopes, significantly stronger immune responses to vaccine candidates can be achieved. In a vaccine candidate designed based on the HIV-1 envelope glycoprotein, researchers have taken a more sophisticated approach by selectively eliminating N-glycans proximal to a conserved epitope recognized by receptor CD4 [16]. This targeted glycoengineering successfully elicited broadly neutralizing antibodies directed at the CD4 binding site.

By modifying the interfaces of virus–host interaction, glycans play a Jekyll and Hyde role in viral infection. The sites and structures of glycans in prevailing viral strains as well as the recognition pattern of glycan-binding proteins (viral or host) are likely the result of co-evolution between viruses and their hosts. Manipulating the host glycosylation process has the potential to tilt the balance to win the antiviral battle. However, there are several challenges along this path. Firstly, the heterogeneity of glycosylation driven by non-template biosynthesis makes it difficult to accurately determine the structure–function relationship. It is particularly challenging to analyse the functional glycome of viral surface proteins because it differs in different host cells, as described above. Previous studies have consistently demonstrated that the glycosylation patterns of soluble recombinant envelope proteins deviate significantly from those of authentic viruses. This disparity complicates the task of elucidating the functional role of the viral glycome. Secondly, computational tools are currently lacking for accurately modeling the 3D glycome information displayed on viral surfaces. While biochemical methods, such as glycan microarrays, are frequently used to screen the affinity of glycan-binding proteins towards synthetic oligosaccharides, such methods hardly represent the real interaction surface between virus and host because they do not take into account the arrangement of glycan ligands on virions. As glycan-binding proteins heavily rely on the avidity of glycan ligands to compensate for their relatively low affinity, computational tools are essential for accurately modeling the 3D glycan information displayed on viral surfaces. Finally, methods are needed to extrapolate evolutionary changes of viral glycosylations from longitudinal analyses of viral strains in history. These methods can help in predicting the potential changes of viral glycosylation in the future, thus guiding our understanding of changes in viral infectivity, host range and immune recognition. Last but not least, the biosynthetic machinery of glycans significantly diverges between evolutionally distant hosts. Thus, distinct rules may govern glycan-mediated viral–host interactions in different kingdoms. For example, the unique glycan structures of plants may play completely different roles in the infection of phytoviruses—an area that remains poorly studied.

Many of the challenges mentioned above are inherent limitations in current glycobiology studies. Similarly to nucleic acids and proteins, glycans are indispensable biological macromolecules. However, their complexity presents unique challenges in dissecting protein–glycan interactions. The efforts to characterize glycan ligands that mediate viral–host interactions, monitor glycan changes during virus–host co-evolution and ultimately model functional glycans on virions offer an ideal biological framework for deciphering the biological information encoded by glycan chains, often referred to as the ‘glyco-code’.

## Supplementary Material

nwae086_Supplemental_File
