# Supplementary material for: Evolving roles of glycosylation in the tug-of-war between virus and host
Source: Natl Sci Rev. 2024 Mar 7;11(5):nwae086. doi: 10.1093/nsr/nwae086 (PMC11114464; doi:10.1093/nsr/nwae086)
Supplement: nwae086_Supplemental_File [file nwae086_supplemental_file.docx]

**Supplemental Table 1 Summary of major types of glycans that can be recognized by virus as attachment factors or as entry receptors**

| **Virus family** | **Species** | **Virus** | | **Glycan type/structure** | **Reference** |
| --- | --- | --- | --- | --- | --- |
| Adenoviridae | Unclassified Human adenoviruses | HAdV | Human adenovirus | HS | [1] |
|  | Unclassified Human adenoviruses | HAdV | Human adenovirus | SA | [2] |
| Caliciviridae | [Norwalk virus](https://www.ncbi.nlm.nih.gov/Taxonomy/Browser/wwwtax.cgi?mode=Tree&id=11983&lvl=3&lin=f&keep=1&srchmode=1&unlock) | NoV | Norovirus | HBGA | [3] |
|  | Recovirus A | TV | Tulane Virus | HBGA | [4] |
| Coronaviridae | Betacoronavirus 1 | HCoV-OC43 | Human Coronavirus OC43 | SA | [5] |
|  | Human coronavirus HKU1 | HKU1 | Human Coronavirus HKU1 | SA | [5] |
|  | Human coronavirus NL63 | HCoV-NL63 | Human Coronavirus NL63 | HS | [6] |
|  | Middle East respiratory syndrome-related coronavirus | MERS-CoV | Middle East Respiratory Syndrome Coronavirus | SA | [7] |
|  | Middle East respiratory syndrome-related coronavirus | MERS-CoV | Middle East Respiratory Syndrome Coronavirus | HS | [8] |
|  | Severe acute respiratory syndrome-related coronavirus | SARS CoV-2 | Severe Acute Respiratory Syndrome Coronavirus 2 | HS | [9-11] |
|  | Severe acute respiratory syndrome-related coronavirus | SARS CoV | Severe Acute Respiratory Syndrome Coronavirus | HS | [12] |
|  | Severe acute respiratory syndrome-related coronavirus | SARS CoV-2 | Severe Acute Respiratory Syndrome Coronavirus 2 | SA | [13-16] |
| Filoviridae | Orthomarburgvirus marburgense | MARV | Marburg Virus | HS | [17] |
|  | Orthoebolavirus zairense | EBOV | Ebola Virus | HS | [18] |
| Flaviviridae | Hepacivirus hominis | HCV | Hepatitis C Virus | HS | [19] |
|  | Orthoflavivirus denguei | DENV | Dengue Virus | HS | [20] |
|  | Orthoflavivirus denguei | DENV | Dengue Virus | SA | [21] |
|  | Orthoflavivirus encephalitidis | TBEV | Tick-Borne Encephalitis Virus | HS | [22] |
|  | Orthoflavivirus flavi | YFV | Yellow Fever Virus | HS | [23] |
|  | Orthoflavivirus japonicum | JEV | Japanese Encephalitis Virus | HS | [24] |
|  | Orthoflavivirus murrayense | MVEV | Murray Valley Encephalitis Virus | HS | [25] |
|  | Orthoflavivirus nilense | WNV | West Nile Virus | HS | [26] |
|  | Orthoflavivirus zikaense | ZIKV | Zika Virus | HS | [27] |
|  | Orthoflavivirus zikaense | ZIKV | Zika Virus | CS | [27] |
|  | Orthoflavivirus zikaense | ZIKV | Zika Virus | SA | [28] |
| Hepadnavirdae | Hepatitis B virus | HBV | Hepatitis B Virus | HS | [29] |
| Hepeviridae | unclassified Orthohepevirus | HEV | Hepatitis E Virus | HS | [30] |
| Orthoherpesviridae | Cytomegalovirus humanbeta5 | HCMV | Human cytomegalovirus | HS | [31] |
|  | Cytomegalovirus humanbeta5 | HCMV | Human Cytomegalovirus | SA | [32] |
|  | Lymphocryptovirus humangamma4 | EBV | Epstein-Barr Virus | HS | [33] |
|  | Rhadinovirus humangamma8 | HHV-8 | Human Herpesvirus 8 | HS | [34] |
|  | Simplexvirus humanalpha1/2 | HSV | Herpes Simplex Virus | HS | [35] |
|  | Varicellovirus humanalpha3 | VZV | Varicella Zoster Virus | HS | [36] |
|  | Roseolovirus humanbeta7 | HHV-7 | Human Herpesvirus 7 | HS | [37] |
| Orthomyxoviridae | Alphainfluenzavirus | IAV | Influenza A Virus | SA | [38-44] |
|  | Betainfluenzavirus | IBV | Influenza B Virus | SA | [45] |
|  | Gammainfluenzavirus | ICV | Influenza C Virus | SA | [46] |
|  | Deltainfluenzavirus | IDV | Influenza D Virus | SA | [47] |
| Papillomaviridae | Human Papillomavirus | HPV | Human Papillomavirus | HS | [48] |
| Paramyxoviridae | Henipavirus hendraense | HeV | Hendra Virus | HS | [49] |
|  | Henipavirus nipahense | NiV | Nipah Virus | HS | [49] |
|  | Orthorubulavirus laryngotracheitidis | HPIV | Human Parainfluenza Virus | HS | [50] |
|  | Orthorubulavirus laryngotracheitidis | HPIV | Human Parainfluenza Virus | SA | [51] |
|  | Orthorubulavirus parotitidis | MuV | Mumps Virus | SA | [52] |
| Parvoviridae | Dependoparvovirus primate1 | AAV2 | Adeno-Associated Virus 2 | HS | [53] |
|  | Dependoparvovirus primate1 | AAV1/4/6 | Adeno-Associated Virus1/4/6 | SA | [54, 55] |
|  | Dependoparvovirus mammalian1 | AAV5 | Adeno-Associated Virus 5 | SA | [55] |
| Phenuiviridae | Phlebovirus napoliense | SFNV | Sandfly Fever Naples Virus | HS | [56] |
|  | Phlebovirus riftense | RVFV | Rift Valley Fever Virus | HS | [57] |
| Picornaviridae | Enterovirus A | EV-A71 | Enterovirus A71 | HS | [58] |
|  | Enterovirus B | CVB3 | Coxsackievirus B3 | HS | [59] |
|  | Enterovirus B | CVA9 | Coxsackievirus A9 | HS | [60] |
|  | Enterovirus C | CV-A24 | Coxsackievirus A24 | SA | [61] |
|  | Enterovirus D | EV-D68 | Enterovirus D68 | SA | [62, 63] |
|  | Enterovirus D | EV70 | Enterovirus 70 | SA | [64] |
|  | Foot-and-mouth disease virus | FMDV | Foot-and-mouth disease virus | HS | [65] |
|  | Rhinovirus A | HRV8 | Human rhinovirus type 8 | HS | [66] |
|  | Rhinovirus A | HRV54 | Human rhinovirus type 54 | HS | [67] |
|  | Rhinovirus A | HRV89 | Human rhinovirus type 89 | HS | [68] |
| Pneumoviridae | Metapneumovirus hominis | hMPV | Human Metapneumovirus | HS | [69] |
|  | Orthopneumovirus bovis | RSV | Respiratory Syncytial Virus | HS | [70] |
| Polyomaviridae | Alphapolyomavirus nonihominis | HpyV9 | Human polyomavirus 9 | SA | [71] |
|  | Alphapolyomavirus quintihominis | MCPyV | Merkel Cell Polyomavirus | SA | [72] |
|  | Betapolyomavirus hominis | BKPyV | BK Polyomavirus | SA | [73] |
|  | Betapolyomavirus macacae | SV40 | Simian virus 40 | SA | [74] |
|  | Betapolyomavirus secuhominis | JCPyV | JC Polyomavirus | SA | [75] |
| Poxviridae | Monkeypox Virus | MPXV | Monkeypox Virus | HS | [76] |
| Spinareoviridae | Mammalian orthoreovirus | MRV | Mammalian orthoreovirus 1 Lang | SA | [77] |
| Retroviridae | Human Immunodeficiency Virus | HIV | Human Immunodeficiency Virus | HS | [78] |
|  | Primate T-lymphotropic virus 1 | HTLV-1 | Human T-Cell Leukemia Virus Type 1 | HS | [79] |
| Rhabdoviridae | Lyssavirus rabies | RABV | Rabies Virus | HS | [80] |
| Sedoreoviridae | Rotavirus A | RVA | Rotavirus A | SA | [81] |
|  | Rotavirus A | RVA | Rotavirus A | HBGA | [82] |
|  | Rotavirus C | RVC | Rotavirus C | HBGA | [83] |
| Togaviridae | Chikungunya Virus | CHIKV | Chikungunya Virus | HS | [84] |
|  | Eastern Equine Encephalitis Virus | EEEV | Eastern Equine Encephalitis Virus | HS | [85] |
|  | Getah Virus | GETV | Getah Virus | HS | [86] |
|  | Ross River Virus | RRV | Ross River Virus | HS | [87] |
|  | Semliki Forest Virus | SFV | Semliki Forest Virus | HS | [88] |
|  | Sindbis Virus | SINV | Sindbis Virus | HS | [89] |
|  | Venezuelan Equine Encephalitis Virus | VEEV | Venezuelan Equine Encephalitis Virus | HS | [90] |

**Supplemental Table 2 Viruses interacting with host cell lectins**

| **Virus family** | **Virus** | | **Host lectins** | **Cell type** | **Functions** | **Reference** |
| --- | --- | --- | --- | --- | --- | --- |
| [Arenaviridae](https://www.ncbi.nlm.nih.gov/Taxonomy/Browser/wwwtax.cgi?mode=Undef&id=11617&lvl=3&keep=1&srchmode=1&unlock) | JUNV | Junin virus | DC-SIGN, L-SIGN | 3T3 cells,Vero cells | Entry | [91] |
| [Arenaviridae](https://www.ncbi.nlm.nih.gov/Taxonomy/Browser/wwwtax.cgi?mode=Undef&id=11617&lvl=3&lin=f&keep=1&srchmode=1&unlock) | LASV | Lassa Virus | DC-SIGN | Dendritic cells | Binding | [92] |
|  |  |  | LSECtin | Jurkat cells | Binding | [93] |
| Arenaviridae | LCMV | Lymphocytic Choriomeningitis Virus | DC-SIGN  LSECtin | Jurkat cells | Infection | [94] |
| Coronaviridae | [MERS-CoV](https://pubmed.ncbi.nlm.nih.gov/37808906/) | Middle east respiratory syndrome coronavirus | DC-SIGN | Dendritic cells | Infection Transmission | [95] |
|  | SARS-CoV | Severe acute respiratory syndrome coronavirus | DC-SIGN | Dendritic cells | Infection Transmission | [96, 97] |
|  |  |  | LSECtin | 293T cells | Infection | [98] |
|  | SARS-CoV-2 | Severe acute respiratory syndrome coronavirus-2 | DC-SIGN | Human endothelial cells | Entry | [99] |
|  |  |  | L-SIGN | Liver sinusoidal endothelial cells | Binding | [100] |
|  | IBV | Infectious bronchitis virus | DC-SIGN, L-SIGN | 3T3 or CRFK cells | Infection | [101] |
| Filoviridae | EBOV | Ebola virus | DC-SIGN | Dendritic cells | Binding, Transmission | [102] |
|  |  |  | L-SIGN | 293T cells | Infection | [103] |
|  |  |  | LSECtin | 293T cells | Binding | [104] |
|  |  |  | MGL | K562 cells | Infection | [105] |
|  |  |  | MBL | THP-1 cells and human monocyte-derived macrophages | Infection | [106] |
|  | MARV | Marburg virus | DC-SIGN, L-SIGN | 293T cells | Infection Transmission | [97] |
|  |  |  | ASGPR | HepG2 cells | Binding | [107] |
| Flaviviridae | DENV | Dengue virus | DC-SIGN | Human immature dendritic cells | Binding, Entry | [108] |
|  |  |  | MR | Human macrophages cells | Binding | [109] |
|  | HCV | Hepatitis C virus | DC-SIGN, L-SIGN | 293T cells | Binding | [110] |
|  |  |  | BDCA-2 | COS-7 cells | Binding | [111] |
|  | JEV | Japanese encephalitis virus | DC-SIGN, L-SIGN, LSECtin | Daudi cells | Binding | [112] |
|  | WNV | [West Nile virus](https://pubmed.ncbi.nlm.nih.gov/20513541/) | DC-SIGN | THP-1 cells | Infection | [113] |
|  |  |  | L-SIGN | K562 cells | Infection | [114] |
|  | ZIKV | Zika Virus | DC-SIGN | Dendritic cells and Langerhans cells | Binding, Transmission | [115] |
| Hepadnavividae | HBV | Hepatitis B virus | ASGPR | HepG2 cells | Binding, Entry | [116] |
| Orthoherpesviridae | HCMV | Human cytomegalovirus | DC-SIGN | Dendritic cells | Binding | [117] |
|  | HSV | Herpes simplex virus | DC-SIGN | Dendritic cells | Binding, Transmission | [118] |
|  |  |  | PILR-a | Human primary cells | Infection | [119] |
|  |  |  | Siglec-4 | HL-60 cells | Infection | [120] |
|  | VZV | Varicella-zoster virus | Siglec-4 | OL cells | Infection | [120] |
| Orthomyxoviridae | IAV | Influenza A virus | DC-SIGN, L-SIGN | Lec2 cells | Infection | [121] |
|  |  |  | MGL, MR | J774E Mφ, RAW 264.7 Mφ (RAW) or LA-4 epithelial cells | Binding | [122] |
| [Paramyxoviridae](https://www.ncbi.nlm.nih.gov/Taxonomy/Browser/wwwtax.cgi?mode=Undef&id=11158&lvl=3&lin=f&keep=1&srchmode=1&unlock) | MV | Measles virus | DC-SIGN | Dendritic cells | Binding, Transmission | [123] |
|  | SeV | Sendai virus | ASGPR | 1-7-1 cells | Binding | [124] |
| Phenuiviridae | PTV | Punta Toro virus | DC-SIGN | Raji cells | Infection | [125] |
|  | RVFV | Rift Valley fever virus | DC-SIGN | Raji cells | Binding | [125] |
|  | SFTSV | Severe Fever with Thrombocytopenia Virus | DC-SIGN | Dendritic cells | Entry | [126] |
|  | TOSV | [Toscana virus](https://pubmed.ncbi.nlm.nih.gov/21914151/) | DC-SIGN | Raji cells | Infection | [125] |
|  | UUKV | Uukuniemi virus | DC-SIGN | HeLa cells and Vero cells | Binding | [125] |
| [Picornaviridae](https://www.ncbi.nlm.nih.gov/Taxonomy/Browser/wwwtax.cgi?mode=Undef&id=12058&lvl=3&lin=f&keep=1&srchmode=1&unlock) | HAV | Hepatitis A virus | ASGPR | HepG2 cell line and primary hepatocytes | Binding, Entry | [127] |
| Retroviridae | HIV | Human immunodeficiency virus | DC-SIGN | T cells | Binding, Infection | [103] |
|  |  |  | L-SIGN | 293T cells | Binding | [128] |
|  |  |  | DCIR | Dendritic cells and CD4 T cells | Infection, Transmission | [129] |
|  |  |  | MR | Sperm cells | Infection, Transmission | [130] |
|  |  |  | Siglec-1 | Dendritic cells | Binding, Infection, Transmission | [131] |
|  |  |  | SP-A | Dendritic cells | Binding | [132] |
|  | HTLV | Human T-cell lymphotropic virus | DC-SIGN | Dendritic cells | Binding, Transmission, Infection | [133] |
|  | SIV | Simian immunodeficiency virus | DC-SIGN | Dendritic cells | Binding, Transmission | [134] |
| [Togaviridae](https://www.ncbi.nlm.nih.gov/Taxonomy/Browser/wwwtax.cgi?mode=Undef&id=11018&lvl=3&keep=1&srchmode=1&unlock) | AURAV | Aura virus | DC-SIGN, L-SIGN | 293T or 3T3 cells | Binding | [135] |
|  | SFV | Semliki Forest Virus | DC-SIGN, L-SIGN | 293T or 3T3 cells | Binding | [136] |
|  | SINV | Sindbis virus | DC-SIGN, L-SIGN | THP-1 cells | Binding | [137] |

Note: DC-SIGN: Dendritic cell-specific intracellular adhesion molecules (ICAM)-3 grabbing non-integrin; L-SIGN: liver/lymph node-specific ICAM-3 grabbing non-integrin; ASGPR: [Asialoglycoprotein receptor](https://pubmed.ncbi.nlm.nih.gov/26000135/); BDCA-2: Blood dendritic cell antigen-2; DCIR: Dendritic cell immunoreceptor; MBL: Mannose-binding lectin; LSECtin: Liver/lymph node sinusoidal endothelial cell C-type lectin; MGL: Macrophage Gal/GalNAc-specific C-type lectin; MR: Mannose receptor; PILR-a: Paired immunoglobulin-like type 2 receptor alpha; SP-A: Surfactant protein A; SP-D: Surfactant protein D

**Supplemental Table 3 Viral proteins modified by O-GlcNAcylation**

| **Virus genus** | **virus** | **Viral protein** | **Reference** |
| --- | --- | --- | --- |
| Adenoviridae | Serotype-2 adenovirus | Fibre Protein | [138] |
| Baculoviridae | Baculovirus | gp41 | [139] |
| Caliciviridae | Norovirus | capsid protein VP1 | [140] |
| Orthoherpesviridae | Kaposi's sarcoma-associated herpesvirus | ORF8, ORF9, ORF2, ORF K3, ORF17, ORF21, ORF25, ORF29, ORF44, ORF K8, ORF54, ORF K10, ORF K11, ORF60, ORF61, ORF63, ORF69 and ORF75 | [141] |
| Orthoherpesviridae | Human Cytomegalovirus | UL32, UL35 | [142, 143] |
| Potyviridae | Plum pox virus | capsid protein (CP) | [144] |
| Sedoreoviridae | Rotavirus | NS26 | [145] |
| Polyomaviridae | Simian virus 40 | large T antigen | [146] |

**Supplemental Table 4 Summary of altered glycosylation affecting vaccine efficacy**

| **Virus genus** | **Virus** | **Vaccine type** | **Change of host cell /Glycoengineering strategy** | **Reference** |
| --- | --- | --- | --- | --- |
| Influenza viruses | H1N1 | Recombinant HA protein | produced in insect cells | [147] |
| Hepatitis viruses | HCV | Recombinant E2 protein | produced in insect cells | [148] |
| Coronavirus | SARS-CoV-2 | Recombinant Spike protein | produced in insect cells | [149, 150] |
| Coronavirus | SARS-CoV-2 | Recombinant Spike protein | produced in yeast | [151] |
| Hepatitis virus | HBV | VLP | produced in mammalian cell lines | [152] |
| Influenza viruses | H3N2 | Inactivated virus | Extending N-glycan | [153] |
| Lentivirus | HIV | Recombinant EnV-based nanoparticle | Glycan trimming | [154] |
| Lentivirus | HIV | Recombinant EnV-based liposome | Eliminating proximal N-glycans | [155] |
| Influenza viruses | H7N9 | Recombinant HA protein | Altered glycosylation sites | [156] |
| Influenza viruses | H1N1 | Recombinant HA protein | Glycan trimming | [157, 158] |
| Influenza viruses | H5N1 | DNA vaccine encoding HA | Ablation of glycosylation sites | [159] |
| Influenza viruses | H5N1 | Recombinant HA protein | Ablation of glycosylation sites | [160] |
| Flavivirus | WNV | Recombinant E and NS1 proteins | Ablation of glycosylation sites | [161] |
| Coronavirus | SARS-CoV-2 | Recombinant Spike protein | Glycan trimming | [162, 163] |
| Ebola virus | EBOV | Recombinant GP -based ferritin nanoparticle | Altered glycosylation sites | [164] |
| Respiratory Syncytial Virus | RSV | Recombinant Fusion Protein | Ablation of glycosylation sites | [165] |

REFERENCES

1. Schroer K, Alshawabkeh M, Schellhorn S *et al.* Influence of Heparan Sulfate Proteoglycans and Factor X on species D Human Adenovirus Uptake and Transduction. *Viruses*. 2022; **15**(1).

2. Chandra N, Frängsmyr L, Imhof S *et al.* Sialic Acid-Containing Glycans as Cellular Receptors for Ocular Human Adenoviruses: Implications for Tropism and Treatment. *Viruses-Basel*. 2019; **11**(5).

3. Cao S, Lou ZY, Tan M *et al.* Structural basis for the recognition of blood group trisaccharides by norovirus. *J Virol*. 2007; **81**(11): 5949-5957.

4. Farkas T, Cross RW, Hargitt E *et al.* Genetic Diversity and Histo-Blood Group Antigen Interactions of Rhesus Enteric Caliciviruses. *J Virol*. 2010; **84**(17): 8617-8625.

5. Li Z, Lang Y, Liu L *et al.* Synthetic O-acetylated sialosides facilitate functional receptor identification for human respiratory viruses. *Nat Chem*. 2021; **13**(5): 496-503.

6. Milewska A, Zarebski M, Nowak P *et al.* Human coronavirus NL63 utilizes heparan sulfate proteoglycans for attachment to target cells. *J Virol*. 2014; **88**(22): 13221-13230.

7. Park YJ, Walls AC, Wang Z *et al.* Structures of MERS-CoV spike glycoprotein in complex with sialoside attachment receptors. *Nat Struct Mol Biol*. 2019; **26**(12): 1151-1157.

8. Hao W, Ma B, Li Z *et al.* Binding of the SARS-CoV-2 spike protein to glycans. *Sci Bull (Beijing)*. 2021; **66**(12): 1205-1214.

9. Clausen TM, Sandoval DR, Spliid CB *et al.* SARS-CoV-2 Infection Depends on Cellular Heparan Sulfate and ACE2. *Cell*. 2020; **183**(4): 1043-1057 e1015.

10. Liu L, Chopra P, Li XR *et al.* Heparan Sulfate Proteoglycans as Attachment Factor for SARSCoV-2. *Acs Central Sci*. 2021; **7**(6): 1009-1018.

11. Bermejo-Jambrina M, Eder J, Kaptein TM *et al.* Infection and transmission of SARS-CoV-2 depend on heparan sulfate proteoglycans. *Embo J*. 2021; **40**(20).

12. Lang J, Yang N, Deng J *et al.* Inhibition of SARS pseudovirus cell entry by lactoferrin binding to heparan sulfate proteoglycans. *PLoS One*. 2011; **6**(8): e23710.

13. Pronker MF, Creutznacher R, Drulyte I *et al.* Sialoglycan binding triggers spike opening in a human coronavirus. *Nature*. 2023; **624**(7990): 201-206.

14. Nguyen L, McCord KA, Bui DT *et al.* Sialic acid-containing glycolipids mediate binding and viral entry of SARS-CoV-2. *Nat Chem Biol*. 2022; **18**(1): 81-90.

15. Tomris I, Unione L, Nguyen L *et al.* SARS-CoV-2 Spike N-Terminal Domain Engages 9-O-Acetylated alpha2-8-Linked Sialic Acids. *ACS Chem Biol*. 2023; **18**(5): 1180-1191.

16. Negi G, Sharma A, Chaudhary M *et al.* SARS-CoV-2 Binding to Terminal Sialic Acid of Gangliosides Embedded in Lipid Membranes. *ACS Infect Dis*. 2023; **9**(7): 1346-1361.

17. O'Hearn A, Wang MX, Cheng H *et al.* Role of EXT1 and Glycosaminoglycans in the Early Stage of Filovirus Entry. *J Virol*. 2015; **89**(10): 5441-5449.

18. Tamhankar M, Gerhardt DM, Bennett RS *et al.* Heparan sulfate is an important mediator of Ebola virus infection in polarized epithelial cells. *Virol J*. 2018; **15**.

19. Xu Y, Martinez P, Seron K *et al.* Characterization of hepatitis C virus interaction with heparan sulfate proteoglycans. *J Virol*. 2015; **89**(7): 3846-3858.

20. Chen Y, Maguire T, Hileman RE *et al.* Dengue virus infectivity depends on envelope protein binding to target cell heparan sulfate. *Nat Med*. 1997; **3**(8): 866-871.

21. Cime-Castillo J, Delannoy P, Mendoza-Hernández G *et al.* Sialic Acid Expression in the Mosquito

and Its Possible Role in Dengue Virus-Vector Interactions. *Biomed Res Int*. 2015; **2015**.

22. Kroschewski H, Allison SL, Heinz FX *et al.* Role of heparan sulfate for attachment and entry of tick-borne encephalitis virus. *Virology*. 2003; **308**(1): 92-100.

23. Germi R, Crance JM, Garin D *et al.* Heparan sulfate-mediated binding of infectious dengue virus type 2 and yellow fever virus. *Virology*. 2002; **292**(1): 162-168.

24. Su CM, Liao CL, Lee YL *et al.* Highly sulfated forms of heparin sulfate are involved in japanese encephalitis virus infection. *Virology*. 2001; **286**(1): 206-215.

25. Lee E, Lobigs M. Mechanism of virulence attenuation of glycosaminoglycan-binding variants of Japanese encephalitis virus and Murray valley encephalitis virus. *J Virol*. 2002; **76**(10): 4901-4911.

26. Lee E, Hall RA, Lobigs M. Common E protein determinants for attenuation of glycosaminoglycan-binding variants of Japanese encephalitis and West Nile viruses. *J Virol*. 2004; **78**(15): 8271-8280.

27. Kim SY, Zhao J, Liu X *et al.* Interaction of Zika Virus Envelope Protein with Glycosaminoglycans. *Biochemistry*. 2017; **56**(8): 1151-1162.

28. Tan CW, Hor CHH, Kwek SS *et al.* Cell surface α2,3-linked sialic acid facilitates Zika virus internalization. *Emerg Microbes Infec*. 2019; **8**(1): 426-437.

29. Schulze A, Gripon P, Urban S. Hepatitis B virus infection initiates with a large surface protein-dependent binding to heparan sulfate proteoglycans. *Hepatology*. 2007; **46**(6): 1759-1768.

30. Kalia M, Chandra V, Rahman SA *et al.* Heparan sulfate proteoglycans are required for cellular binding of the hepatitis E virus ORF2 capsid protein and for viral infection. *J Virol*. 2009; **83**(24): 12714-12724.

31. Neyts J, Snoeck R, Schols D *et al.* Sulfated Polymers Inhibit the Interaction of Human Cytomegalovirus with Cell-Surface Heparan-Sulfate. *Virology*. 1992; **189**(1): 48-58.

32. Lobert PE, Hober D, Dewilde A *et al.* Cell-Membrane Bound N-Acetylneuraminic Acid Is Involved in the Infection of Fibroblasts and Phorbol-Ester Differentiated Monocyte-Like Cells with Human Cytomegalovirus (Hcmv). *Arch Virol*. 1995; **140**(8): 1357-1371.

33. Chesnokova LS, Valencia SM, Hutt-Fletcher LM. The BDLF3 gene product of Epstein-Barr virus, gp150, mediates non-productive binding to heparan sulfate on epithelial cells and only the binding domain of CD21 is required for infection. *Virology*. 2016; **494**: 23-28.

34. Birkmann A, Mahr K, Ensser A *et al.* Cell surface heparan sulfate is a receptor for human herpesvirus 8 and interacts with envelope glycoprotein K8.1. *J Virol*. 2001; **75**(23): 11583-11593.

35. WuDunn D, Spear PG. Initial interaction of herpes simplex virus with cells is binding to heparan sulfate. *J Virol*. 1989; **63**(1): 52-58.

36. Jacquet A, Haumont M, Chellun D *et al.* The varicella zoster virus glycoprotein B (gB) plays a role in virus binding to cell surface heparan sulfate proteoglycans. *Virus Res*. 1998; **53**(2): 197-207.

37. Skrincosky D, Hocknell P, Whetter L *et al.* Identification and analysis of a novel heparin-binding glycoprotein encoded by human herpesvirus 7. *J Virol*. 2000; **74**(10): 4530-4540.

38. Fukuzawa K, Omagari K, Nakajima K *et al.* Sialic acid recognition of the pandemic influenza 2009 H1N1 virus: binding mechanism between human receptor and influenza hemagglutinin. *Protein Pept Lett*. 2011; **18**(5): 530-539.

39. Pappas C, Viswanathan K, Chandrasekaran A *et al.* Receptor specificity and transmission of H2N2 subtype viruses isolated from the pandemic of 1957. *PLoS One*. 2010; **5**(6): e11158.

40. Liu M, Bakker AS, Narimatsu Y *et al.* H3N2 influenza A virus gradually adapts to human-type receptor binding and entry specificity after the start of the 1968 pandemic. *Proc Natl Acad Sci U S A*. 2023; **120**(31): e2304992120.

41. Matrosovich MN, Matrosovich TY, Gray T *et al.* Human and avian influenza viruses target different cell types in cultures of human airway epithelium. *Proc Natl Acad Sci U S A*. 2004; **101**(13): 4620-4624.

42. Sun HL, Pu J, Wei YD *et al.* Highly Pathogenic Avian Influenza H5N6 Viruses Exhibit Enhanced Affinity for Human Type Sialic Acid Receptor and In-Contact Transmission in Model Ferrets. *J Virol*. 2016; **90**(14): 6235-6243.

43. Shi Y, Zhang W, Wang F *et al.* Structures and Receptor Binding of Hemagglutinins from Human-Infecting H7N9 Influenza Viruses. *Science*. 2013; **342**(6155): 243-247.

44. Tzarum N, de Vries RP, Zhu X *et al.* Structure and receptor binding of the hemagglutinin from a human H6N1 influenza virus. *Cell Host Microbe*. 2015; **17**(3): 369-376.

45. Burmeister WP, Ruigrok RWH, Cusack S. The 2.2-a Resolution Crystal-Structure of Influenza-B Neuraminidase and Its Complex with Sialic-Acid. *Embo J*. 1992; **11**(1): 49-56.

46. Rogers GN, Herrler G, Paulson JC *et al.* Influenza-C Virus Uses 9-O-Acetyl-N-Acetylneuraminic Acid as a High-Affinity Receptor Determinant for Attachment to Cells. *J Biol Chem*. 1986; **261**(13): 5947-5951.

47. Liu R, Sreenivasan C, Yu H *et al.* Influenza D virus diverges from its related influenza C virus in the recognition of 9-O-acetylated N-acetyl- or N-glycolyl-neuraminic acid-containing glycan receptors. *Virology*. 2020; **545**: 16-23.

48. Giroglou T, Florin L, Schafer F *et al.* Human papillomavirus infection requires cell surface heparan sulfate. *J Virol*. 2001; **75**(3): 1565-1570.

49. Mathieu C, Dhondt KP, Chalons M *et al.* Heparan sulfate-dependent enhancement of henipavirus infection. *mBio*. 2015; **6**(2): e02427.

50. Bose S, Banerjee AK. Role of heparan sulfate in human parainfluenza virus type 3 infection. *Virology*. 2002; **298**(1): 73-83.

51. Amonsen M, Smith DF, Cummings RD *et al.* Human parainfluenza viruses hPIV1 and hPIV3 bind oligosaccharides with α2-3-linked sialic acids that are distinct from those bound by H5 avian influenza virus hemagglutinin. *J Virol*. 2007; **81**(15): 8341-8345.

52. Kubota M, Takeuchi K, Watanabe S *et al.* Trisaccharide containing α2,3-linked sialic acid is a receptor for mumps virus. *P Natl Acad Sci USA*. 2016; **113**(41): 11579-11584.

53. Summerford C, Samulski RJ. Membrane-associated heparan sulfate proteoglycan is a receptor for adeno-associated virus type 2 virions. *J Virol*. 1998; **72**(2): 1438-1445.

54. Huang LY, Patel A, Ng R *et al.* Characterization of the Adeno-Associated Virus 1 and 6 Sialic Acid Binding Site. *J Virol*. 2016; **90**(11): 5219-5230.

55. Kaludov N, Brown KE, Walters RW *et al.* Adeno-associated virus serotype 4 (AAV4) and AAV5 both require sialic acid binding for hemagglutination and efficient transduction but differ in sialic acid linkage specificity. *J Virol*. 2001; **75**(15): 6884-6893.

56. Chianese A, Zannella C, Palma F *et al.* Melittin-Related Peptides Interfere with Sandfly Fever Naples Virus Infection by Interacting with Heparan Sulphate. *Microorganisms*. 2023; **11**(10).

57. de Boer SM, Kortekaas J, de Haan CAM *et al.* Heparan Sulfate Facilitates Rift Valley Fever Virus Entry into the Cell. *J Virol*. 2012; **86**(24): 13767-13771.

58. Kobayashi K, Mizuta K, Koike S. Heparan sulfate attachment receptor is a major selection factor for attenuated enterovirus 71 mutants during cell culture adaptation. *PLoS Pathog*. 2020; **16**(3): e1008428.

59. Zautner AE, Jahn B, Hammerschmidt E *et al.* N- and 6-O-sulfated heparan sulfates mediate internalization of coxsackievirus B3 variant PD into CHO-K1 cells. *J Virol*. 2006; **80**(13): 6629-6636.

60. Merilahti P, Karelehto E, Susi P. Role of Heparan Sulfate in Cellular Infection of Integrin-Binding Coxsackievirus A9 and Human Parechovirus 1 Isolates. *Plos One*. 2016; **11**(1).

61. Mistry N, Inoue H, Jamshidi F *et al.* Coxsackievirus A24 variant uses sialic acid-containing O-linked glycoconjugates as cellular receptors on human ocular cells. *J Virol*. 2011; **85**(21): 11283-11290.

62. Imamura T, Okamoto M, Nakakita S *et al.* Antigenic and Receptor Binding Properties of Enterovirus 68. *J Virol*. 2014; **88**(5): 2374-2384.

63. Liu Y, Sheng J, Baggen J *et al.* Sialic acid-dependent cell entry of human enterovirus D68. *Nat Commun*. 2015; **6**.

64. Nokhbeh MR, Hazra S, Alexander DA *et al.* Enterovirus 70 binds to different glycoconjugates containing α2,3-linked sialic acid on different cell lines. *J Virol*. 2005; **79**(11): 7087-7094.

65. O'Donnell V, LaRocco M, Baxt B. Heparan sulfate-binding foot-and-mouth disease virus enters cells via caveola-mediated endocytosis. *J Virol*. 2008; **82**(18): 9075-9085.

66. Khan AG, Pickl-Herk A, Gajdzik L *et al.* Entry of a heparan sulphate-binding HRV8 variant strictly depends on dynamin but not on clathrin, caveolin, and flotillin. *Virology*. 2011; **412**(1): 55-67.

67. Khan AG, Pichler J, Rosemann A *et al.* Human rhinovirus type 54 infection via heparan sulfate is less efficient and strictly dependent on low endosomal pH. *J Virol*. 2007; **81**(9): 4625-4632.

68. Vlasak M, Goester I, Blaas D. Human rhinovirus type 89 variants use heparan sulfate proteoglycan for cell attachment. *J Virol*. 2005; **79**(10): 5963-5970.

69. Adamson P, Thammawat S, Muchondo G *et al.* Diversity in glycosaminoglycan binding amongst hMPV G protein lineages. *Viruses*. 2012; **4**(12): 3785-3803.

70. Shi D, He P, Song Y *et al.* Interactions of heparin with key glycoproteins of human respiratory syncytial virus. *Front Mol Biosci*. 2023; **10**: 1151174.

71. Khan ZM, Liu Y, Neu U *et al.* Crystallographic and glycan microarray analysis of human polyomavirus 9 VP1 identifies N-glycolyl neuraminic acid as a receptor candidate. *J Virol*. 2014; **88**(11): 6100-6111.

72. Neu U, Hengel H, Blaum BS *et al.* Structures of Merkel Cell Polyomavirus VP1 Complexes Define a Sialic Acid Binding Site Required for Infection. *Plos Pathogens*. 2012; **8**(7).

73. Dugan AS, Eash S, Atwood WJ. An N-linked glycoprotein with α-(2,3)-linked sialic acid is a receptor for BK virus. *J Virol*. 2005; **79**(22): 14442-14445.

74. Campanero-Rhodes MA, Smith A, Chai W *et al.* N-glycolyl GM1 ganglioside as a receptor for simian virus 40. *J Virol*. 2007; **81**(23): 12846-12858.

75. Liu CK, Wei G, Atwood WJ. Infection of glial cells by the human polyomavirus JC is mediated by an N-linked glycoprotein containing terminal α(2-6)-linked sialic acids. *J Virol*. 1998; **72**(6): 4643-4649.

76. Shi DL, He P, Song YF *et al.* Kinetic and Structural Aspects of Glycosaminoglycan-Monkeypox Virus Protein A29 Interactions Using Surface Plasmon Resonance. *Molecules*. 2022; **27**(18).

77. Helander A, Silvey KJ, Mantis NJ *et al.* The viral σ1 protein and glycoconjugates containing α2-3-linked sialic acid are involved in type 1 reovirus adherence to M cell apical surfaces. *J Virol*. 2003; **77**(14): 7964-7977.

78. Roderiquez G, Oravecz T, Yanagishita M *et al.* Mediation of human immunodeficiency virus type 1 binding by interaction of cell surface heparan sulfate proteoglycans with the V3 region of envelope gp120-gp41. *J Virol*. 1995; **69**(4): 2233-2239.

79. Jones KS, Fugo K, Petrow-Sadowski C *et al.* Human T-cell leukemia virus type 1 (HTLV-1) and HTLV-2 use different receptor complexes to enter T cells. *J Virol*. 2006; **80**(17): 8291-8302.

80. Sasaki M, Anindita PD, Ito N *et al.* The Role of Heparan Sulfate Proteoglycans as an Attachment Factor for Rabies Virus Entry and Infection. *J Infect Dis*. 2018; **217**(11): 1740-1749.

81. Haselhorst T, Fleming FE, Dyason JC *et al.* Sialic acid dependence in rotavirus host cell invasion. *Nat Chem Biol*. 2009; **5**(2): 91-93.

82. Hu L, Crawford SE, Czako R *et al.* Cell attachment protein VP8* of a human rotavirus specifically interacts with A-type histo-blood group antigen. *Nature*. 2012; **485**(7397): 256-259.

83. Sun X, Wang L, Qi J *et al.* Human Group C Rotavirus VP8*s Recognize Type A Histo-Blood Group Antigens as Ligands. *J Virol*. 2018; **92**(11).

84. Tanaka A, Tumkosit U, Nakamura S *et al.* Genome-Wide Screening Uncovers the Significance of N-Sulfation of Heparan Sulfate as a Host Cell Factor for Chikungunya Virus Infection. *J Virol*. 2017; **91**(13).

85. Gardner CL, Ebel GD, Ryman KD *et al.* Heparan sulfate binding by natural eastern equine encephalitis viruses promotes neurovirulence. *P Natl Acad Sci USA*. 2011; **108**(38): 16026-16031.

86. Wang NN, Zhai XF, Li XL *et al.* Attenuation of Getah Virus by a Single Amino Acid Substitution at Residue 253 of the E2 Protein that Might Be Part of a New Heparan Sulfate Binding Site on Alphaviruses. *J Virol*. 2022; **96**(6).

87. Kesari AS, Sharkey CM, Sanders DA. Role of heparan sulfate in entry and exit of Ross River virus glycoprotein-pseudotyped retroviral vectors. *Virology*. 2019; **529**: 177-185.

88. Smit JM, Waarts BL, Kimata K *et al.* Adaptation of alphaviruses to heparan sulfate: Interaction of Sindbis and Semliki Forest viruses with liposomes containing lipid-conjugated heparin. *J Virol*. 2002; **76**(20): 10128-10137.

89. Klimstra WB, Ryman KD, Johnston RE. Adaptation of Sindbis virus to BHK cells selects for use of heparan sulfate as an attachment receptor. *J Virol*. 1998; **72**(9): 7357-7366.

90. Bernard KA, Klimstra WB, Johnston RE. Mutations in the E2 glycoprotein of Venezuelan equine encephalitis virus confer heparan sulfate interaction, low morbidity, and rapid clearance from blood of mice. *Virology*. 2000; **276**(1): 93-103.

91. Martinez MG, Bialecki MA, Belouzard S *et al.* Utilization of human DC-SIGN and L-SIGN for entry and infection of host cells by the New World arenavirus, Junin virus. *Biochem Bioph Res Co*. 2013; **441**(3): 612-617.

92. Goncalves AR, Moraz ML, Pasquato A *et al.* Role of DC-SIGN in Lassa Virus Entry into Human Dendritic Cells. *J Virol*. 2013; **87**(21): 11504-11515.

93. Shimojima M, Ströher U, Ebihara H *et al.* Identification of Cell Surface Molecules Involved in Dystroglycan-Independent Lassa Virus Cell Entry. *J Virol*. 2012; **86**(4): 2067-2078.

94. Shimojima M, Kawaoka Y. Cell Surface Molecules Involved in Infection Mediated by Lymphocytic Choriomeningitis Virus Glycoprotein. *J Vet Med Sci*. 2012; **74**(10): 1363-1366.

95. Labiod N, Luczkowiak J, Tapia MM *et al.* The role of DC-SIGN as a trans-receptor in infection by MERS-CoV. *Front Cell Infect Mi*. 2023; **13**.

96. Yang ZY, Huang Y, Ganesh L *et al.* pH-dependent entry of Severe acute respiratory syndrome coronavirus is mediated by the spike glycoprotein and enhanced by dendritic cell transfer through DC-SIGN. *J Virol*. 2004; **78**(11): 5642-5650.

97. Marzi A, Gramberg T, Simmons G *et al.* DC-SIGN and DC-SIGNR interact with the glycoprotein of Marburg virus and the S protein of severe acute respiratory syndrome coronavirus. *J Virol*. 2004; **78**(21): 12090-12095.

98. Gramberg T, Hofmann H, Möller P *et al.* LSECtin interacts with filovirus glycoproteins and the spike protein of SARS coronavirus. *Virology*. 2005; **340**(2): 224-236.

99. Amraei R, Yin WQ, Napoleon MA *et al.* CD209L/L-SIGN and CD209/DC-SIGN Act as Receptors for SARS-CoV-2. *Acs Central Sci*. 2021; **7**(7): 1156-1165.

100. Kondo Y, Larabee JL, Gao L *et al.* L-SIGN is a receptor on liver sinusoidal endothelial cells for SARS-CoV-2 virus. *Jci Insight*. 2021; **6**(14).

101. Zhang YT, Buckles E, Whittaker GR. Expression of the C-type lectins DC-SIGN or L-SIGN alters host cell susceptibility for the avian coronavirus, infectious bronchitis virus. *Vet Microbiol*. 2012; **157**(3-4): 285-293.

102. Alvarez CP, Lasala F, Carrillo J *et al.* C-type lectins DC-SIGN and L-SIGN mediate cellular entry by Ebola virus in cis and in trans. *J Virol*. 2002; **76**(13): 6841-6844.

103. Lin G, Simmons G, Pöhlmann S *et al.* Differential N-linked glycosylation of human immunodeficiency virus and Ebola virus envelope glycoproteins modulates interactions with DC-SIGN and DC-SIGNR. *J Virol*. 2003; **77**(2): 1337-1346.

104. Powlesland AS, Fisch T, Taylor ME *et al.* A novel mechanism for LSECtin binding to Ebola virus surface glycoprotein through truncated glycans. *J Biol Chem*. 2008; **283**(1): 593-602.

105. Fujihira H, Usami K, Matsuno K *et al.* A Critical Domain of Ebolavirus Envelope Glycoprotein Determines Glycoform and Infectivity. *Sci Rep-Uk*. 2018; **8**.

106. Brudner M, Karpel M, Lear C *et al.* Lectin-Dependent Enhancement of Ebola Virus Infection via Soluble and Transmembrane C-type Lectin Receptors. *Plos One*. 2013; **8**(4).

107. Becker S, Spiess M, Klenk HD. The Asialoglycoprotein Receptor Is a Potential Liver-Specific Receptor for Marburg Virus. *J Gen Virol*. 1995; **76**: 393-399.

108. Liu P, Ridilla M, Patel P *et al.* Beyond attachment: Roles of DC-SIGN in dengue virus infection. *Traffic*. 2017; **18**(4): 218-231.

109. Miller JL, Dewet BJM, Martinez-Pomares L *et al.* The mannose receptor mediates dengue virus infection of macrophages. *Plos Pathogens*. 2008; **4**(2).

110. Pöhlmann S, Zhang J, Baribaud F *et al.* Hepatitis C virus glycoproteins interact with DC-SIGN and DC-SIGNR. *J Virol*. 2003; **77**(7): 4070-4080.

111. Florentin J, Aouar B, Dental C *et al.* HCV glycoprotein E2 is a novel BDCA-2 ligand and acts as an inhibitor of IFN production by plasmacytoid dendritic cells. *Blood*. 2012; **120**(23): 4544-4551.

112. Shimojima M, Takenouchi A, Shimoda H *et al.* Distinct usage of three C-type lectins by Japanese encephalitis virus: DC-SIGN, DC-SIGNR, and LSECtin. *Arch Virol*. 2014; **159**(8): 2023-2031.

113. Martina BE, Koraka P, van den Doel P *et al.* DC-SIGN enhances infection of cells with glycosylated West Nile virus in vitro and virus replication in human dendritic cells induces production of IFN-alpha and TNF-alpha. *Virus Res*. 2008; **135**(1): 64-71.

114. Davis CW, Nguyen HY, Hanna SL *et al.* West Nile virus discriminates between DC-SIGN and DC-SIGNR for cellular attachment and infection. *J Virol*. 2006; **80**(3): 1290-1301.

115. Eder J, Zijlstra-Willems E, Koen G *et al.* Transmission of Zika virus by dendritic cell subsets in skin and vaginal mucosa. *Front Immunol*. 2023; **14**.

116. Owada T, Matsubayashi K, Sakata H *et al.* Interaction between desialylated hepatitis B virus and asialoglycoprotein receptor on hepatocytes may be indispensable for viral binding and entry. *J Viral Hepatitis*. 2006; **13**(1): 11-18.

117. Halary F, Amara A, Lortat-Jacob H *et al.* Human cytomegalovirus binding to DC-SIGN is required for dendritic cell infection and target cell trans-infection. *Immunity*. 2002; **17**(5): 653-664.

118. de Jong MAWP, de Witte L, Bolmstedt A *et al.* Dendritic cells mediate herpes simplex virus infection and transmission through the C-type lectin DC-SIGN. *J Gen Virol*. 2008; **89**: 2398-2409.

119. Satoh T, Arii J, Suenaga T *et al.* PILRα is a herpes simplex virus-1 entry coreceptor that associates with glycoprotein B. *Cell*. 2008; **132**(6): 935-944.

120. Suenaga T, Satoh T, Somboonthum P *et al.* Myelin-associated glycoprotein mediates membrane fusion and entry of neurotropic herpesviruses. *P Natl Acad Sci USA*. 2010; **107**(2): 866-871.

121. Gillespie L, Roosendahl P, Ng WC *et al.* Endocytic function is critical for influenza A virus infection via DC-SIGN and L-SIGN. *Sci Rep-Uk*. 2016; **6**.

122. Upham JP, Pickett D, Irimura T *et al.* Macrophage Receptors for Influenza A Virus: Role of the Macrophage Galactose-Type Lectin and Mannose Receptor in Viral Entry. *J Virol*. 2010; **84**(8): 3730-3737.

123. Mesman AW, de Vries RD, McQuaid S *et al.* A prominent role for DC-SIGN+ dendritic cells in initiation and dissemination of measles virus infection in non-human primates. *PLoS One*. 2012; **7**(12): e49573.

124. Leyrer S, Bitzer M, Lauer U *et al.* Sendai virus-like particles devoid of haemagglutinin-neuraminidase protein infect cells via the human asialoglycoprotein receptor. *J Gen Virol*. 1998; **79**: 683-687.

125. Lozach PY, Kühbacher A, Meier R *et al.* DC-SIGN as a Receptor for Phleboviruses. *Cell Host & Microbe*. 2011; **10**(1): 75-88.

126. Hofmann H, Li XX, Zhang XA *et al.* Severe Fever with Thrombocytopenia Virus Glycoproteins Are Targeted by Neutralizing Antibodies and Can Use DC-SIGN as a Receptor for pH-Dependent Entry into Human and Animal Cell Lines. *J Virol*. 2013; **87**(8): 4384-4394.

127. Dotzauer A, Gebhardt U, Bieback K *et al.* Hepatitis A virus-specific immunoglobulin a mediates infection of hepatocytes with hepatitis A virus via the asialoglycoprotein receptor. *J Virol*. 2000; **74**(23): 10950-10957.

128. Geijtenbeek TB, Kwon DS, Torensma R *et al.* DC-SIGN, a dendritic cell-specific HIV-1-binding protein that enhances trans-infection of T cells. *Cell*. 2000; **100**(5): 587-597.

129. Lambert AA, Gilbert C, Richard M *et al.* The C-type lectin surface receptor DCIR acts as a new attachment factor for HIV-1 in dendritic cells and contributes to trans- and cis-infection pathways. *Blood*. 2008; **112**(4): 1299-1307.

130. Cardona-Maya W, Velilla PA, Montoya CJ *et al.* human immunodeficiency virus and sperm cell interaction mediated by the mannose receptor. *J Reprod Immunol*. 2011; **92**(1-2): 1-7.

131. Ruffin N, Gea-Mallorquí E, Brouiller F *et al.* Constitutive Siglec-1 expression confers susceptibility to HIV-1 infection of human dendritic cell precursors. *P Natl Acad Sci USA*. 2019; **116**(43): 21685-21693.

132. Gaiha GD, Dong T, Palaniyar N *et al.* Surfactant protein A binds to HIV and inhibits direct infection of CD4+ cells, but enhances dendritic cell-mediated viral transfer. *J Immunol*. 2008; **181**(1): 601-609.

133. Jain P, Manuel SL, Khan ZK *et al.* DC-SIGN Mediates Cell-Free Infection and Transmission of Human T-Cell Lymphotropic Virus Type 1 by Dendritic Cells. *J Virol*. 2009; **83**(21): 10908-10921.

134. Pöhlmann S, Baribaud F, Lee B *et al.* DC-SIGN interactions with human immunodeficiency virus type 1 and 2 and simian immunodeficiency virus. *J Virol*. 2001; **75**(10): 4664-4672.

135. Froelich S, Tai A, Kennedy K *et al.* Pseudotyping Lentiviral Vectors with Aura Virus Envelope Glycoproteins for DC-SIGN-Mediated Transduction of Dendritic Cells. *Hum Gene Ther*. 2011; **22**(10): 1281-1291.

136. Froelich S, Tai A, Kennedy K *et al.* Virus-Receptor Mediated Transduction of Dendritic Cells by Lentiviruses Enveloped with Glycoproteins Derived from Semliki Forest Virus. *Plos One*. 2011; **6**(6).

137. Klimstra WB, Nangle EM, Smith MS *et al.* DC-SIGN and L-SIGN can act as attachment receptors for alphaviruses and distinguish between mosquito cell- and mammalian cell-derived viruses. *J Virol*. 2003; **77**(22): 12022-12032.

138. Cailletboudin ML, Strecker G, Michalski JC. O-Linked Glcnac in Serotype-2 Adenovirus Fiber. *Eur J Biochem*. 1989; **184**(1): 205-211.

139. Whitford M, Faulkner P. A Structural Polypeptide of the Baculovirus Autographa-Californica Nuclear Polyhedrosis-Virus Contains O-Linked N-Acetylglucosamine. *J Virol*. 1992; **66**(6): 3324-3329.

140. Hanisch FG. Recombinant norovirus capsid protein VP1 (GII.4) expressed in H5 insect cells exhibits post-translational modifications with potential impact on lectin activity and vaccine design. *Glycobiology*. 2022; **32**(6): 496-505.

141. Jochmann R, Pfannstiel J, Chudasama P *et al.* O-GlcNAc transferase inhibits KSHV propagation and modifies replication relevant viral proteins as detected by systematic O-GlcNAcylation analysis. *Glycobiology*. 2013; **23**(10): 1114-1130.

142. Greis KD, Gibson W, Hart GW. Site-specific glycosylation of the human cytomegalovirus tegument basic phosphoprotein (UL32) at serine 921 and serine 952. *J Virol*. 1994; **68**(12): 8339-8349.

143. Salsman J, Jagannathan M, Paladino P *et al.* Proteomic Profiling of the Human Cytomegalovirus UL35 Gene Products Reveals a Role for UL35 in the DNA Repair Response. *J Virol*. 2012; **86**(2): 806-820.

144. Perez Jde J, Udeshi ND, Shabanowitz J *et al.* O-GlcNAc modification of the coat protein of the potyvirus Plum pox virus enhances viral infection. *Virology*. 2013; **442**(2): 122-131.

145. Gonzalez SA, Burrone OR. Rotavirus Ns26 Is Modified by Addition of Single O-Linked Residues of N-Acetylglucosamine. *Virology*. 1991; **182**(1): 8-16.

146. Medina L, Grove K, Haltiwanger RS. SV40 large T antigen is modified with O-linked N-acetylglucosamine but not with other forms of glycosylation. *Glycobiology*. 1998; **8**(4): 383-391.

147. An YM, Parsons LM, Jankowska E *et al.* -Glycosylation of Seasonal Influenza Vaccine Hemagglutinins: Implication for Potency Testing and Immune Processing. *J Virol*. 2019; **93**(2).

148. Li D, von Schaewen M, Wang X *et al.* Altered Glycosylation Patterns Increase Immunogenicity of a Subunit Hepatitis C Virus Vaccine, Inducing Neutralizing Antibodies Which Confer Protection in Mice. *J Virol*. 2016; **90**(23): 10486-10498.

149. Fernandes B, Castro R, Bhoelan F *et al.* Insect Cells for High-Yield Production of SARS-CoV-2 Spike Protein: Building a Virosome-Based COVID-19 Vaccine Candidate. *Pharmaceutics*. 2022; **14**(4).

150. Li T, Zheng Q, Yu H *et al.* SARS-CoV-2 spike produced in insect cells elicits high neutralization titres in non-human primates. *Emerg Microbes Infect*. 2020; **9**(1): 2076-2090.

151. Liu B, Yin Y, Liu Y *et al.* A Vaccine Based on the Receptor-Binding Domain of the Spike Protein Expressed in Glycoengineered Pichia pastoris Targeting SARS-CoV-2 Stimulates Neutralizing and Protective Antibody Responses. *Engineering (Beijing)*. 2022; **13**: 107-115.

152. Joe CCD, Chatterjee S, Lovrecz G *et al.* Glycoengineered hepatitis B virus-like particles with enhanced immunogenicity. *Vaccine*. 2020; **38**(22): 3892-3901.

153. Kikuchi C, Antonopoulos A, Wang S *et al.* Glyco-engineered MDCK cells display preferred receptors of H3N2 influenza absent in eggs used for vaccines. *Nat Commun*. 2023; **14**(1): 6178.

154. Zhang YN, Paynter J, Antanasijevic A *et al.* Single-component multilayered self-assembling protein nanoparticles presenting glycan-trimmed uncleaved prefusion optimized envelope trimmers as HIV-1 vaccine candidates. *Nat Commun*. 2023; **14**(1): 1985.

155. Dubrovskaya V, Tran K, Ozorowski G *et al.* Vaccination with Glycan-Modified HIV NFL Envelope Trimer-Liposomes Elicits Broadly Neutralizing Antibodies to Multiple Sites of Vulnerability. *Immunity*. 2019; **51**(5): 915-929 e917.

156. Kim JI, Park S, Bae JY *et al.* Glycosylation generates an efficacious and immunogenic vaccine against H7N9 influenza virus. *PLoS Biol*. 2020; **18**(12): e3001024.

157. Chen JR, Yu YH, Tseng YC *et al.* Vaccination of monoglycosylated hemagglutinin induces cross-strain protection against influenza virus infections. *Proc Natl Acad Sci U S A*. 2014; **111**(7): 2476-2481.

158. Tseng YC, Wu CY, Liu ML *et al.* Egg-based influenza split virus vaccine with monoglycosylation induces cross-strain protection against influenza virus infections. *Proc Natl Acad Sci U S A*. 2019; **116**(10): 4200-4205.

159. Bright RA, Ross TM, Subbarao K *et al.* Impact of glycosylation on the immunogenicity of a DNA-based influenza H5 HA vaccine. *Virology*. 2003; **308**(2): 270-278.

160. Liu WC, Jan JT, Huang YJ *et al.* Unmasking Stem-Specific Neutralizing Epitopes by Abolishing N-Linked Glycosylation Sites of Influenza Virus Hemagglutinin Proteins for Vaccine Design. *J Virol*. 2016; **90**(19): 8496-8508.

161. Whiteman MC, Li L, Wicker JA *et al.* Development and characterization of non-glycosylated E and NS1 mutant viruses as a potential candidate vaccine for West Nile virus. *Vaccine*. 2010; **28**(4): 1075-1083.

162. Huang HY, Liao HY, Chen X *et al.* Vaccination with SARS-CoV-2 spike protein lacking glycan shields elicits enhanced protective responses in animal models. *Sci Transl Med*. 2022; **14**(639): eabm0899.

163. Chen WH, Wei JF, Kundu RT *et al.* Genetic modification to design a stable yeast-expressed recombinant SARS-CoV-2 receptor binding domain as a COVID-19 vaccine candidate. *Bba-Gen Subjects*. 2021; **1865**(6).

164. Xu D, Powell AE, Utz A *et al.* Design of universal Ebola virus vaccine candidates via immunofocusing. *bioRxiv*. 2023.

165. Leemans A, Boeren M, Van der Gucht W *et al.* Removal of the N-Glycosylation Sequon at Position N116 Located in p27 of the Respiratory Syncytial Virus Fusion Protein Elicits Enhanced Antibody Responses after DNA Immunization. *Viruses-Basel*. 2018; **10**(8).
